# The Importance of Recognising Mild Cases of Diphtheria

**Published:** 1904-01-09

**Authors:** 


### The Importance of Recognising Mild Forms of Diphtheria.

At the present moment methods of preventative medicine are receiving a large share of notice, not only from the members of the profession but from the laity also, a fact which may be noted with much satisfaction. It is perhaps a sign of the times that especial attention should be thus bestowed upon the wider issues of medicine—to the relations that exist between diseases and communities rather than to the more limited field of enterprise that offers itself in the consideration of illness affecting individuals; although this does not mean that there has been, or ought to be, any relaxation of endeavour in the study of the latter aspect. Very far from it; the healing art, as distinct from the science of prevention of disease, was never before in such a flourishing condition. But State medicine is without question the highest department of the profession, and it is only right that proper recognition should be given to this fact, so that any suggestions which may be put forward by experts for the safeguarding of the public health may receive all the serious consideration which they merit. The prevention of such diseases as can be prevented, or at any rate which can be held within reasonable bounds, is a duty incumbent on the Government of every country, and adequate legislation has already done incalculable service to humanity in this direction. It is equally true that much more remains to be achieved. The general prevalence of pulmonary tuberculosis, of infantile diarrhœa, and of enteric fever might almost with certainty be lessened by the putting in force of suitable measures backed up by the might and authority of the law. The same is true with regard to diphtheria. From the annual report of the Local Government Board for 1902 we learn that there were notified during that year in England and Wales 33,231 cases of diphtheria, the death-rate among these cases being 14 per cent., so that 4,804 deaths were due to this disease alone. Moreover, knowing what we do with regard to the age incidence of diphtheria, it may be assumed that the majority of the deaths occurred in young people and not in those of an age when life's work has been partly or wholly accomplished; this means a correspondingly greater loss to the nation. Although the notification and isolation of this affection is compulsory by law, not every case of diphtheria which comes under the notice of general practitioners is notified and isolated. Very far from it. This is a case where theory and practice do not go hand in hand, and the stumbling-block is to be found in the mild cases, where the patients, while not displaying the typical clinical symptoms with the formation of membrane and so forth, yet harbour virulent micro-organisms in their respiratory passages and may communicate the disease in a severe form to individuals who are more susceptible to infection than themselves, and in whom a fatal result may ensue. A case in point occurred some years ago in one of our large public schools. Several cases of diphtheria occurred among the boys. On endeavouring to ascertain the source of infection, the medical officer of the institution noted that nearly all the cases occurred among the boys of one dormitory and one particular form. Now there were two boys who slept in this dormitory and who were in the affected form. On examining these two boys, one of them was found to have a nasal discharge, and it was remarked by his fellows that latterly he had snored at night, a habit that he had not indulged in before. This boy was forthwith isolated and no further cases of diphtheria occurred in the school. There could be no reasonable doubt that the nasal catarrh from which this boy suffered was diphtherial in nature and was a source of danger to others.

What is required is a proper recognition of these mild cases of diphtheria and the provision of statutory power to notify them and have the patients isolated. It is even now a moot point as to whether cases should be notified as diphtheria which do not give the clinical evidences of the severe forms of that illness. We still remember, with some feeling of uneasiness, the remonstrances and vituperation which were hurled against us by a general practitioner and by the mother of a family because we notified one of her children, who had been brought to a London hospital as an out-patient, as suffering from diphtheria on the ground that it had a sore throat from which diphtheria bacilli were obtained, while a case of diphtheria had occurred in the next house a few days previously. What is really necessary in the interests of the public is specific authorisation and command for medical practitioners to notify the mild cases of sore throat which occur so frequently in the course of epidemics, provided that bacterial examination shows the presence of diphtheria bacilli.

